# From light to sound: Seeing and hearing the placenta in health and disease

**DOI:** 10.1126/sciadv.aed2184

**Published:** 2026-01-28

**Authors:** Donghyun Lee, Jiwoong Kim, Jinseok Heo, Hyunseo Jeon, Shiyang Chang, Wonseok Choi, Chulhong Kim, Zhifen Yang

**Affiliations:** ^1^ Departments of Convergence IT Engineering, Electrical Engineering, Mechanical Engineering, and Medical Science and Engineering, POSTECH-CATHOLIC Biomedical Engineering Institute, Medical Device Innovation Center, Pohang University of Science and Technology, Pohang, Republic of Korea.; ^2^ Department of Histology and Embryology, Hebei Medical University, Shijiazhuang, China.; ^3^ Department of Biomedical Engineering and Medical Sciences, College of Medicine, The Catholic University of Korea, Seoul, Republic of Korea.; ^4^ Opticho Inc., Pohang, Republic of Korea.; ^5^ Department of Obstetrics, The Fourth Hospital of Hebei Medical University, Shijiazhuang, China.

## Abstract

The placenta is a pregnancy-specific organ, functioning as the maternal-fetal interface and mediating essential processes including exchange, protection, and endocrine regulation. Placental abnormalities contribute to the pathophysiology, onset, progression, and prognosis of major perinatal disorders, making imaging modalities that enable their detection and monitoring crucial. Ultrasound (US) imaging is the principal modality for placental imaging, providing structural assessment with B-mode and blood flow evaluation with Doppler mode. Furthermore, advanced techniques such as 3D power Doppler, quantitative US, and shear-wave elastography expand the capabilities of placental US imaging. Photoacoustic (PA) imaging enables observation of the optical properties of endogenous chromophores (e.g., hemoglobin) with high spatial resolution and measures blood oxygenation, a key factor in placental dysfunction, offering substantial value for placental imaging. In this review, we cover US/PA imaging techniques for placental imaging, including preclinical and clinical studies. In addition, by considering current limitations and potential solutions, we suggest future trajectories for the advancement of US/PA imaging in this field.

## Teaser

US and PA imaging provide structural and functional placental assessment, serving as key modalities for maternal-fetal management.

## INTRODUCTION

**Fig. 1.**
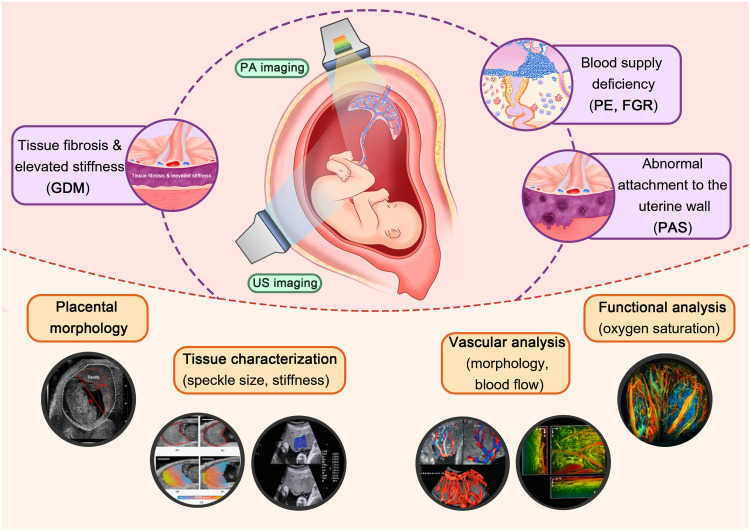
Overview of perinatal disorders attributable to placental abnormalities and imaging approaches. This figure was created using Adobe Photoshop, with certain elements adapted from (*71*) [CC BY (https://creativecommons.org/licenses/by/4.0/)], (*74*) [CC BY-NC-ND (https://creativecommons.org/licenses/by-nc-nd/4.0/)], (*94*) (reused with permission), (*103*) [CC BY-NC (https://creativecommons.org/licenses/by-nc/4.0/)], (*118*) [CC BY (https://creativecommons.org/licenses/by/4.0/)], (*147*) (reused with permission).

The placenta is a transient yet vital organ in pregnancy, acting as the life-support interface between the mother and the fetus. It mediates gas exchange, nutrient transfer, and endocrine regulation and provides immune protection to the developing fetus (*1*). The functional and structural integrity of the placenta is therefore crucial for both maternal and fetal health. Placental dysfunction and structural alterations are strongly associated with major pregnancy complications such as preeclampsia (PE), fetal growth restriction (FGR), placenta accreta spectrum (PAS), gestational diabetes mellitus (GDM), preterm birth, and recurrent miscarriage (Fig. 1) (*2*–*4*). PE refers to a pregnancy-specific hypertensive disorder characterized by new-onset hypertension after 20 weeks of gestation with proteinuria or maternal organ dysfunction, whereas FGR denotes a condition in which the fetus fails to achieve its genetically determined growth potential in utero due to pathological growth restriction. PAS is characterized by abnormal placental villous adherence or invasion into the myometrium and, in severe cases, extension into adjacent pelvic organs. GDM is a pregnancy-associated metabolic disorder defined by glucose intolerance that arises or is first diagnosed during pregnancy in women without preexisting diabetes. These perinatal disorders are associated with increased perinatal morbidity and mortality and are commonly linked to adverse maternal and fetal outcomes. Therefore, there is an urgent need for noninvasive, high-resolution, and dynamic imaging technologies that can enable early detection, accurate diagnosis, and timely intervention for placental abnormalities.

Now, ultrasound (US) imaging is the standard for placental and fetal monitoring in clinical practice due to its real-time imaging capability and safety. US imaging visualizes overall placental anatomy and estimates blood flow in the uteroplacental and fetoplacental circulations, such as through uterine artery (UtA) and umbilical artery (UA) Doppler, facilitating the screening of impaired placentation. Further, advanced US-based techniques have been reported to improve diagnostic and predictive performance by advancing conventional B-mode and Doppler imaging and by integrating them with serum biomarkers or complementary imaging modalities. US techniques such as three-dimensional (3D) power Doppler for visualization and quantification of placental vascular structures, as well as quantitative US and shear-wave elastography (SWE) for quantifying placental tissue characteristics, have been explored. In parallel, photoacoustic (PA) imaging provides superior contrast for chromophores like hemoglobin, relying on the PA effect, which converts absorbed light energy into US waves (*5*–*8*). This unique capability allows PA imaging to capture functional information, such as blood oxygenation, alongside high-resolution vascular anatomy, offering insights into placental hypoxia, which is a key indicator of placental dysfunction (*9*–*12*). Combining PA and US imaging leverages their complementary strengths: high spatial resolution and deep tissue penetration of US imaging for anatomical context and functional and molecular sensitivity of PA imaging for physiological information (*13*–*23*).

In this review, we aim to provide a comprehensive overview of the research landscape surrounding US and PA imaging for placental and fetal studies. We discuss how these technologies have been applied to investigate placental structure, blood flow, and oxygenation, and we highlight the remaining challenges in effectively translating them into routine clinical practice. Last, we outline future directions and opportunities for leveraging US and PA imaging as robust, noninvasive tools to improve placental health monitoring and pregnancy outcomes.

## IMAGING MODALITIES: PRINCIPLES AND PERFORMANCE

### Conventional imaging techniques: Ultrasound and magnetic resonance imaging

US imaging has been an irreplaceable, routine modality for OB/GYN clinics thanks to its accessibility and high spatiotemporal resolution for fast on-site diagnosis of placental structure and function (*24*). Fundamentally, US pulse waves travel through tissue and are reflected back to the imaging probe according to acoustic impedance differences between propagation media. The amplitude and frequency components of the received US signal are used to reconstruct B-mode images of tissue structures and Doppler measurements of blood flow, respectively.

B-mode US is primarily used to assess structural information, including placental location (anterior, posterior, low-lying, or previa), placental thickness, and the degree of calcification (Grannum grading). Typically, the placenta appears as a hyperechoic band surrounding the chorionic sac from ~10 weeks of gestation and becomes clearly distinguishable after 14 weeks (*25*). As pregnancy advances, the previously homogeneous echogenicity of the placental parenchyma and chorionic plate becomes heterogeneous, with the development of calcifications that form the basis for Grannum grading. Transvaginal US in early gestation (around 6 weeks) can visualize the gestational sac and early trophoblast-decidua complex. As the placenta begins to take shape after ~10 weeks, its position and early morphology can be more reliably assessed. Then, transabdominal US is typically used to evaluate placental position, size, thickness, and internal echotexture after ~10 weeks.

However, although B-mode US enables staging of placental maturity, placental grading offers a limited predictive value for placental function, and related disorders such as PE, FGR, PAS, and GDM remain beyond the diagnostic scope of this technique. Consequently, Doppler US techniques (e.g., pulsed-wave, color, and power Doppler) are essential for quantitatively evaluating blood flow velocity and vascular resistance in the UtA and UA, thereby assessing placental hemodynamic function, which underlies maternal-fetal exchange (*26*). Doppler mode may allow differentiation between maternal and fetal blood flow within the placenta and may indicate placental perfusion deficiency through indices such as the UA pulsatility index (PI).

In cases where US results are inconclusive or additional soft-tissue characterization is required, magnetic resonance imaging (MRI) can serve as a complementary modality (*27*). MRI uses a strong static magnetic field (typically 1.5 to 3 T) to prealign all protons longitudinally and spatiotemporally varying magnetic field gradients (~mT) to excite and deflect the protons toward the transverse plane. During their realignment back to the longitudinal direction, the induced magnetic signal undergoes two relaxation processes—T1 and T2—that differ depending on tissue composition.

MRI is capable of both structural and functional assessment of soft tissues. For vascular imaging, fast MRI sequences are designed to capture the phase contrast in magnetization between flowing blood and stationary tissue, enabling measurement of blood flow direction and velocity. Diffusion-weighted (DW) MRI can sensitively detect the degree of water molecule diffusion within tissues, providing additional tissue contrast and perfusion information (*28*). Blood oxygen level–dependent MRI imaging estimates hemoglobin oxygen saturation (sO_2_) by exploiting the paramagnetic property of deoxyhemoglobin. For placental evaluation, MRI is generally avoided during the first trimester due to potential risk to the developing embryo. While 3-T MRI provides higher spatial resolution and contrast, 1.5-T MRI is preferred to minimize potential fetal risk. The more sensitive assessment of perfusion can be achieved using gadolinium-based contrast agents, which, however, are not practical due to the risk of fetotoxicity (*29*). Contrast-free functional MRI techniques such as DW MRI can help delineate the placenta-myometrium interface to aid in the detection of myometrial invasion (*30*).

### Novel imaging techniques: Advanced US and PA imaging

With advancements in computing power of US imaging systems, planewave-based ultrafast US imaging has brought a breakthrough in high-resolution US vascular imaging (*31*). Planewave imaging acquires the entire field of view (FOV) at thousands of frames per second, several orders of magnitude faster than conventional imaging modes, and coherent compounding of angle-steered acquisitions improves image quality.

Planewave-based power Doppler imaging, often referred to as ultrafast Doppler (UFD) or functional US, has been introduced as a novel contrast-free, high-resolution vascular imaging technique. An ensemble of hundreds of compounded planewave US frames is transformed into a spatiotemporal Casorati matrix, followed by an SVD-based clutter filtering that surpasses conventional temporal clutter filtering (*32*). After SVD clutter filtering, each frame containing moving speckles (ideally corresponding to blood cells) is power-summed to reconstruct the power Doppler vascular image, where pixel intensity represents blood flow volume. Existing US-based assessments of placental circulation have predominantly focused on two major vessels: the UtA, reflecting maternal hemodynamics, and the UA, reflecting fetal circulatory status. In contrast, UFD leverages its superior vessel detection sensitivity to directly assess intraplacental blood flow, an aspect increasingly recognized as critical for risk prediction, diagnosis, and clinical management of perinatal disorders.

Instead of endogenous blood cells, microbubble contrast agents can be deployed to enhance the sensitivity and clarity of vascular flow imaging, thereby enabling a super-resolution technique referred to as US localization microscopy (ULM) (*33*). Microbubbles are injected at a low concentration to ensure that only a countable and separable number of microbubbles exist within local regions of interest. This enables the localization process, which detects local intensity maxima corresponding to sparse speckle signals from microbubbles that stand out from the dense speckle background produced by endogenous blood cells. The collection of localized pixels provides super-resolution vascular morphology and flow velocities via speckle tracking. For placental imaging, however, it remains unclear whether microbubbles can penetrate the placental barrier; to the best of our knowledge, there have been no reports yet on ULM of the placenta.

PA imaging, a hybrid acoustic-optical modality, enables high-resolution visualization and quantitative assessment of placental vascular architecture and sO_2_, emerging as a promising tool in placental research. PA imaging uses ultrasonic signals generated by target tissue in response to pulsed laser irradiation, analogous to “thunder after lightning” (*34*–*36*). The amplitude of the PA signal represents the magnitude of photothermal volume expansion, which is proportional to the product of the optical absorption coefficient, local optical fluence, and the optical-to-thermal energy conversion efficiency. In placental research, PA tomography (PAT) is particularly advantageous, as its deep tissue sensitivity and volumetric imaging capability allow comprehensive 3D assessment of the placenta. The key advantage of PAT lies in the spectroscopic differentiation of tissue components using multiple optical wavelengths (*37*, *38*). Such rich optical contrast is reconstructed as a high-resolution ultrasonic image at depths extending beyond the optically ballistic regime, reaching several centimeters. In particular, hemoglobin exhibits strong optical absorption in the near-infrared (NIR) range, where oxy- and deoxy-hemoglobin are spectroscopically distinguishable, enabling sO_2_ measurement for functional assessment (*39*–*41*). Placental sO_2_ serves as a critical indicator of fetal oxygen supply, closely linked to fetal survival and development, and can reveal placental insufficiency associated with the pathophysiology of perinatal disorders. PAT enables concurrent assessment of sO_2_ and high-resolution vascular anatomy, supporting early diagnosis and risk stratification of perinatal disorders. In addition, leveraging its strong molecular sensitivity, PAT can incorporate exogenous contrast agents to provide additional information on specific tissues and structures (e.g., tumors, lymphatics, and the fetus), as well as molecular targets and physiological or pathological processes beyond those assessed with endogenous absorbers alone (*5*, *10*).

PA imaging can be implemented in a microscopic form known as PA microscopy (PAM) (*42*–*51*). Two variations of PAM exist; acoustic-resolution PAM and optical-resolution PAM (OR-PAM), depending on whether the spatial resolution is determined by acoustic of optical focusing (*52*). Specifically, OR-PAM is implemented in a more optically dominated regime, using tightly focused laser illumination with optional acoustic focusing. Compared to optical microscopy techniques, OR-PAM benefits from depth-resolved signal detection, enabling 3D structural visualization from a single raster scan. Spectroscopic PAM is also possible with a limited selection of optical wavelengths (by means such as multiple laser sources or stimulated Raman scattering), although the spatial resolution varies with wavelength. However, PAM exhibits a substantially shallower imaging depth compared with PAT and therefore is generally limited to investigating superficial structures or requires the use of abdominal imaging windows to access placental tissues. OR-PAM can be applied for intravital imaging of superficial tissues such as skin or through optical windows to visualize subsurface organs. By resolving capillary-level structures and performing spectroscopic measurements of hemoglobin states, PAM allows precise assessment of microvascular remodeling and localized oxygenation dynamics that are not accessible with the deeper-penetrating but lower-resolution PAT.

### Performance comparison

**Fig. 2.**
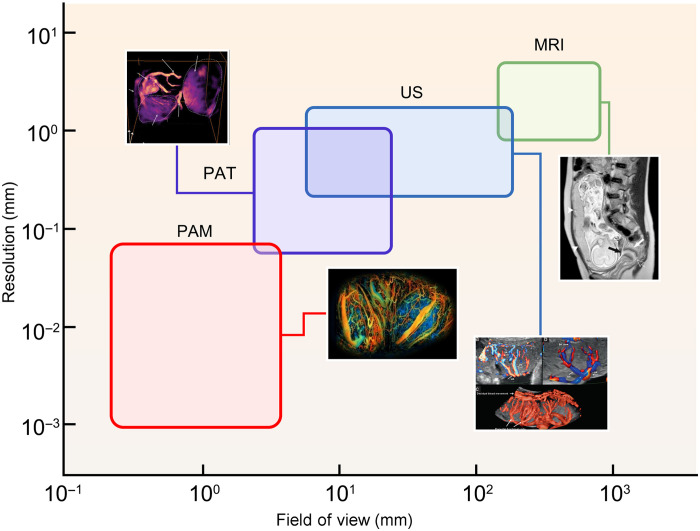
Performance summary of placental imaging modalities. Figures were reproduced from (*103*) [CC BY-NC (https://creativecommons.org/licenses/by-nc/4.0/)], (*104*) [CC BY (https://creativecommons.org/licenses/by/4.0/)], (*147*, *148*) (reused with permission).

Figure 2 benchmarks the typical imaging performances of each placental imaging modality, showing the general tradeoff between FOV and spatial resolution. Conventional clinical modalities—MRI and US—are capable of whole-body imaging of the fetus and placenta with millimeter to submillimeter resolution. For preclinical research, PAT, PAM, and high-frequency US imaging can image the fetus and placenta in small animal models with micrometer- scale to several-tens-of-micrometers resolution. US and PA modalities provide placental visualization across a wide range of scales, from micrometer-scale preclinical targets to centimeter-scale clinical targets, holding promise for translational research from benchside to bedside.

## ANIMAL MODELS FOR PLACENTAL STUDY

Since most human placental research relies on tissues obtained after delivery, the ability to understand dynamic variations during pregnancy remains limited. To overcome these constraints, animal models serve as essential preclinical platforms. As emphasized in initiatives such as the “Human Placenta Project,” these preclinical studies play an indispensable role in establishing new imaging biomarkers and validating the safety and effectiveness of emerging technologies for clinical use (*53*).

### Principal perinatal diseases and pathophysiology

**Fig. 3.**
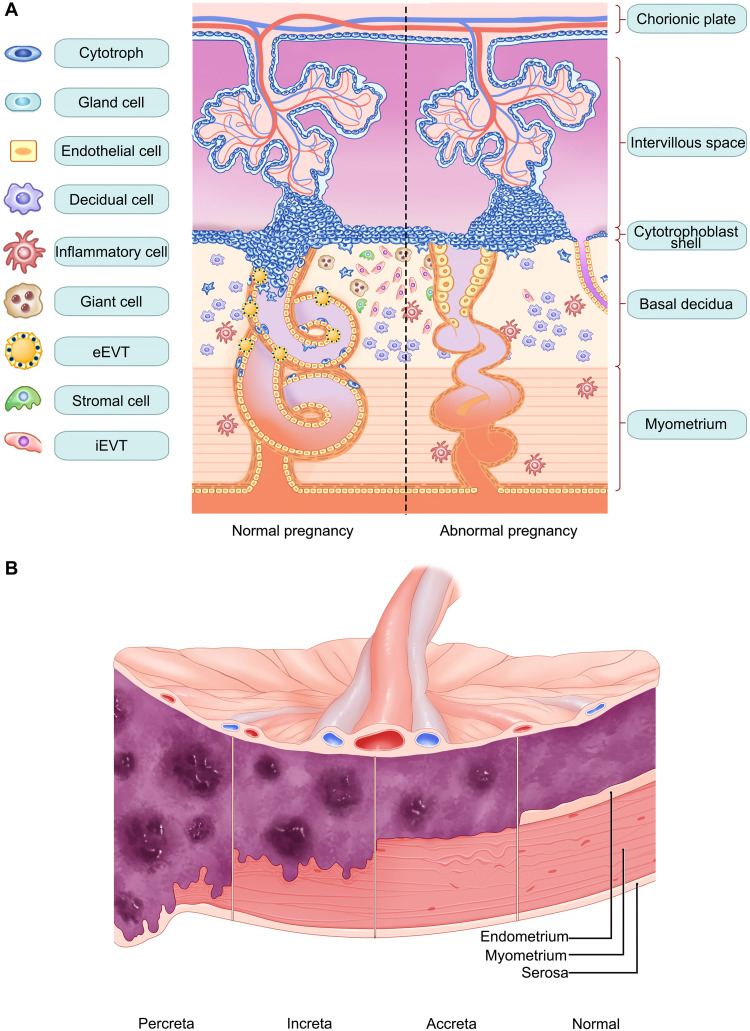
Schematic overview of abnormal placental development in major perinatal disorders. (**A**) Normal versus abnormal spiral artery remodeling: In normal pregnancy, extravillous trophoblasts remodel spiral arteries into dilated, low-resistance vessels. In PE, FGR, etc., insufficient invasion leads to incomplete remodeling, higher vascular resistance, and reduced placental perfusion. eEVT, endovascular extravillous trophoblasts; iEVT, interstitial extravillous trophoblasts. (**B**) Depth of placental invasion: In normal pregnancy, villi are confined to the decidua. In PAS, accreta denotes villi attached to the myometrium, increta penetrates into the myometrium, and percreta traverses the myometrium and may reach the bladder. (A) was partially adapted from our previously published work (*149*) [CC BY-NC-ND (https://creativecommons.org/licenses/by-nc-nd/4.0/)], and (A) and (B) were created using Adobe Photoshop.

The pathophysiology of major perinatal disorders such as PE, FGR, PAS, and GDM is closely linked to placental structure and function. PE and FGR are characterized by inadequate trophoblast invasion, defective spiral artery remodeling, placental hypoperfusion, and impaired oxygen and nutrient delivery. This pathophysiology arises in the first trimester, whereas clinical symptoms typically emerge in the second or third trimester. PAS, comprising placenta accreta, increta, and percreta, represents a major cause of postpartum hemorrhage, making early diagnosis crucial. The fundamental pathophysiology of PAS involves abnormal trophoblastic invasion, characterized by direct extension of chorionic villi into the myometrium and, in severe cases, toward the bladder, due to the absence of the decidua basalis. GDM is characterized by hyperglycemia that arises or is first diagnosed during pregnancy in women without preexisting diabetes. The primary cause is increased insulin resistance after the second trimester, driven by placental and maternal hormones (e.g., placental lactogen, progesterone, cortisol, prolactin, and growth hormone) that impair insulin action; GDM arises when pancreatic β cells fail to compensate. GDM can induce placental changes, including increased vascular resistance, tissue fibrosis, and elevated tissue stiffness. Together, these perinatal disorders converge on the placenta as a central pathogenic hub, where aberrant trophoblast invasion and spiral artery remodeling result in compromised uteroplacental circulation. A schematic overview is presented in Fig. 3.

### Major animal models for placental research

Among rodents, mice are the most commonly used model because of their ease of genetic modification, short gestational period, and low cost (*54*). Mice share a hemochorial placental structure with humans, in which fetal-derived trophoblast cells are in direct contact with maternal blood, making this a key similarity for studying nutrient and gas exchange. Compared with mice, rats are more suitable for surgical procedures and allow the collection of larger tissue and blood sample volumes. Guinea pigs have a comparatively longer gestational period, and their pups are precocial at birth, making them well suited for studies of late gestation. Furthermore, placental structure and hormone profiles are notably similar to those of humans, making guinea pigs sufficiently applicable for human-related studies (*55*).

Rabbits have a hemodichorial placenta, which resembles the human hemomonochorial placenta, and their relatively large body size and distinct placental band formation make them useful for establishing surgical procedures and implementing imaging protocols. They have also played an important role in developing and validating advanced imaging techniques, such as maternal-fetal blood flow differentiation using UFD and real-time oxygen saturation monitoring using PA imaging in a hypoxia model (*56*, *57*).

Sheep are widely used in studies of fetal physiology, owing to their large fetal size and long gestational period. A notable advantage is that standard clinical practices for FGR monitoring can be directly replicated with clinical US machines (*58*). However, because the epitheliochorial placenta is separated by multiple layers, maternal and fetal tissues are not in direct contact. This fundamental difference from the human placenta poses a limitation, making sheep unsuitable for research on invasive trophoblastic diseases (*55*).

Nonhuman primates have the placental structures most similar to those of humans, particularly regarding the depth of trophoblast invasion and spiral artery remodeling, and are therefore considered the most suitable animal models for clinical translation (*59*). Nevertheless, their use is restricted by high costs and ethical concerns.

### Pregnancy complication models

To model PE, the reduced uterine perfusion pressure (RUPP) and the N^G^-nitro-l-arginine methyl ester (L-NAME) administration models are widely used. The RUPP model is a surgical approach that induces placental ischemia by placing clips on the abdominal aorta and UAs during mid-gestation to mimic the clinical features of human PE (*60*). Following surgery, maternal blood pressure increases in late gestation, accompanied by proteinuria, inducing a chronic ischemic state in the placenta (*61*). In maternal circulation, endothelial dysfunction is characterized by elevated sFlt-1 and sEng levels, decreased vascular endothelial growth factor, placental growth factor levels, and reduced fetal weight. Although RUPP has rarely been applied to mice due to microsurgical challenges, a recently developed clip-free ligation method targeting the ovarian and uterine vessels enables induction of a PE-like phenotype. The L-NAME model replicates the systemic effects of vasoconstriction and hypertension by administering the nitric oxide synthase inhibitor L-NAME to pregnant animals. This induces maternal hypertension, proteinuria, elevated sFlt-1 levels, and impaired growth of the placenta and fetus (*62*). Offspring from the L-NAME administration model exhibit growth restriction at birth, followed by neurodevelopmental abnormalities (e.g., delayed motor development and impaired learning and memory). This model not only reproduces maternal symptoms but also enables investigation of long-term effects on subsequent generations.

FGR is characterized by factors such as alterations in placental blood flow, impaired nutrient transport, and structural abnormalities of the placenta. A representative experimental model of FGR involves restricting maternal food intake during pregnancy (*63*). In a nutrient-restricted environment, the placenta exhibits a reduced surface area for nutrient exchange, along with an increase in the thickness of the interhemal membrane, the barrier between maternal and fetal blood. These structural alterations reduce the efficiency of nutrient transport and lead to dysregulation of the insulin-like growth factor (IGF) and glucocorticoid systems. Increased expression of IGF binding proteins, which function as growth inhibitors, impairs placental development, while elevated corticosterone levels in both maternal and fetal blood interfere with fetal organ maturation. UA ligation is also a well-established model of FGR, induced by partially tying both UAs to reduce uteroplacental perfusion (*64*). Offspring from the UA ligation model exhibit metabolic syndrome-like symptoms in adulthood, including impaired glucose tolerance, hyperinsulinemia, and dyslipidemia. Similarly, maternal exposure to a hypoxic environment during pregnancy can impair placental vascular development and consequently restrict fetal growth (*65*).

PAS models are developed by surgically injuring the endometrium and myometrium of mice in a nonpregnant state, followed by induction of pregnancy, thereby generating a local environment conductive to abnormal trophoblast invasion (*66*). As a result, the PAS mouse model exhibits both excessive invasion of extravillous trophoblasts and immune dysregulation at the maternal-fetal interface. Androgen excess models, in which testosterone or dihydrotestosterone is administered during early pregnancy, are used to investigate changes in placental development under a hormonal environment resembling polycystic ovary syndrome (*67*). Models that disrupt maternal immune tolerance through immunoregulatory abnormalities are used to analyze their impact on placental immune cell composition and the maintenance of pregnancy (*68*).

GDM models are reproduced by inducing maternal obesity and insulin resistance through a high-fat/high-sugar diet, combined with administration of streptozotocin in early gestation to partially impair pancreatic β cell function (*69*). The GDM animals exhibited elevated fasting blood glucose, reduced insulin secretion (reflected by decreased C-peptide levels), and abnormal lipid profiles, accompanied by weight gain and hyperglycemia. In this model, the placentas are heavier than those of controls, and the fetuses also exhibit macrosomia, reflecting fetal overgrowth associated with maternal overnutrition observed in GDM. Moreover, the increased expression of inflammatory cytokines [interleukin-6 (IL-6) and tumor necrosis factor–α] and the reduced antioxidant capacity in the placenta indicate that abnormalities in glucose metabolism trigger stress responses within the placental microenvironment (*70*).

## ULTRASOUND TECHNIQUES FOR PLACENTA IMAGING

### Preclinical studies

Placental US imaging is predominantly conducted in clinical research, focusing on major perinatal diseases. However, preclinical research provides distinct advantages, including the elucidation of pathophysiological mechanisms, validation of emerging techniques, and correlation with histological and molecular analyses. This section is organized into three technical domains: advanced hemodynamic assessment, quantitative US, and high-resolution microvascular imaging.

**Fig. 4.**
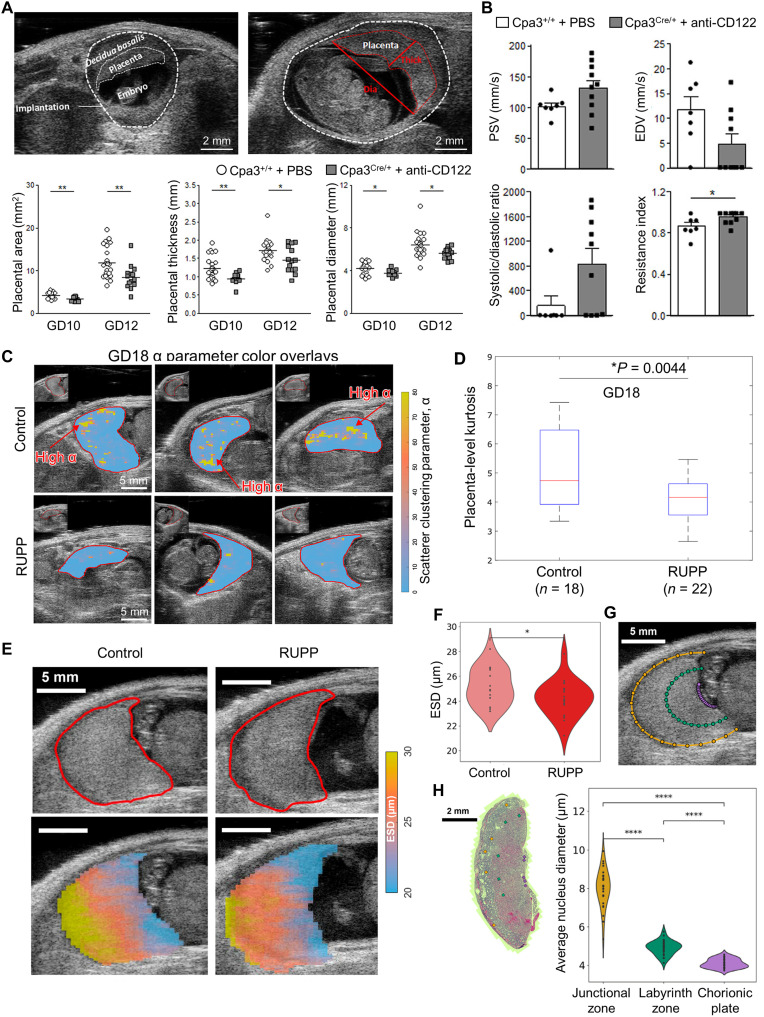
Preclinical studies using placental US imaging. (**A**) Representative US images of WT implantations at GD10 and GD12 and quantitative comparisons of placental dimensions between WT Cpa3^+/+^ + phosphate-buffered saline (PBS) mice (*n* = 3 to 5; placentas = 12 to 22 per day) and MC/NK-deficient Cpa3Cre/^+^ + anti-CD122 mice (*n* = 3 to 4; placentas = 8 to 14 per day) at GD10 and 12. Data are shown as individual values for each placenta with mean. Statistical differences were determined by unpaired *t* test (*P < 0.05 and **P < 0.01). WT, wild type; thick, thickness; dia, diameter. (**B**) Quantitative analysis of the UA at GD14 in Cpa3^+/+^ + PBS mice (*n* = 3; UA measurements = 7) and Cpa3Cre^/+^ + anti-CD122 mice (*n* = 3; UA measurements = 10). Data are presented as mean ± SEM. Statistical analysis was performed using an unpaired *t* test (*P < 0.05). PSV, peak systolic velocity. (**C**) Representative α-overlaid placental images of control and RUPP model at GD18. (**D**) Placenta-level kurtosis box plots at GD18. (**E**) B-mode images and effective scatterer diameter (ESD) parameter maps of control and RUPP model placentas. (**F**) Violin plot comparing average ESD values between control (*n* = 18) and RUPP placentas (*n* = 20); *P < 0.05. (**G**) Placenta sampling curves. (**H**) Hematoxylin and eosin image showing the junctional zone (gold), labyrinth zone (green), and chorionic plate (purple), together with a violin plot comparing cell nucleus diameters across regions for all placentas (*n* = 27) and a scatterplot correlating nucleus diameter with ESD values. Figures are adapted from (*71*) [CC BY (https://creativecommons.org/licenses/by/4.0/)], (*73*) (reused with permission), (*74*) [CC BY-NC-ND (https://creativecommons.org/licenses/by-nc-nd/4.0/)].

In rodent models, high-frequency US enables longitudinal assessment of placental function. Meyer *et al.* (*71*) demonstrated that high-frequency US combined with Doppler imaging can elucidate the progression of FGR. They simultaneously depleted uterine natural killer (NK) and peripheral NK cells in mast cell (MC)–deficient (Cpa3^Cre^/+^^) mice by injecting an anti-CD122 antibody. Using a high-frequency US system (VisualSonics Vevo 2100), they performed assessments at gestational day 5 (GD5), GD8, GD10, GD12, and GD14, demonstrating the occurrence of FGR. At GD10 and GD12, implantation size and placental dimensions were significantly reduced in the NK/MC-deficient group (Fig. 4A). By GD14, reductions in placental thickness and fetal weight persisted in the NK/MC-deficient group, and a reduced feto-placental index indicated impaired placental function. At GD14, UA Doppler showed decreased end-diastolic velocity (EDV), increased resistive index (RI), and an elevated systolic/diastolic (S/D) ratio in the NK/MC-deficient group (Fig. 4B). In some fetuses, absent or reversed end-diastolic flow was observed.

To address the limited sensitivity of UA Doppler PI in detecting mild FGR, Rahman *et al.* (*72*) proposed a US technique to address the limited sensitivity of UA Doppler PI in detecting mild FGR. They applied wave reflection theory, hypothesizing that the UA waveform comprises a forward wave generated by the fetal heart and a reflected wave returning from the placental vessels. They measured UA diameter (via M-mode) and blood flow velocity (via pulsed-wave Doppler) to decompose the waveform and further quantified the reflection coefficient, time delay, and dispersion of the reflected wave. The method was used in CD1 and C57BL/6 mice to evaluate the ability to distinguish placental vascular differences. At embryonic day 17.5 (E17.5), the reflection coefficient and UA PI were 33 and 21% higher, respectively, in C57BL/6 mice than in CD1 mice. Receiver operating characteristic (ROC) analysis yielded area under the curve (AUC) values of 0.94 and 0.83, respectively, demonstrating that the reflection coefficient was more sensitive than UA PI.

In addition to hemodynamics, quantitative US, which is already widely used in clinical practice, has also been applied. Gleed *et al.* (*73*) sought to capture tissue microstructural alterations in PE by using a homodyne K-distribution (HKD) model, which characterizes US speckle statistics and represents diverse scattering conditions. Baseline scans were performed on nine pregnant Sprague-Dawley rats at GD14, and five subsequently underwent RUPP surgery. At GD18, 20 placentas from the RUPP group and 18 from the control group were analyzed. Using the LZ250 probe (13 to 24 MHz) coupled with the Vevo2100 system, 100 radiofrequency (RF) echo frames were acquired over 4 s. The parameters α (scatterer clustering) and κ (coherent-to-diffuse power ratio) were estimated using HKD fitting. Subsequent temporal analysis included visualization, kurtosis assessment, and frame-level statistical evaluation. In the GD18 comparison, the RUPP group exhibited lower values of both α and kurtosis than the control group (Fig. 4, C and D). Markel *et al.* (*74*) also aimed to quantify placental tissue microstructural characteristics in PE rats. The effective scatterer diameter (ESD) was derived by fitting a Gaussian scattering model to the backscatter coefficient obtained from RF data. RUPP surgery was performed on GD14 in five of the nine Sprague-Dawley rats. At GD18, RF data were acquired from 20 RUPP and 18 control placentas using the Vevo2100 system equipped with the LZ250 probe. Overall placental ESD was 1.0 μm smaller in the RUPP group (Fig. 4, E and F). At GD19, 27 placentas were dissected and analyzed histologically. Following hematoxylin and eosin staining, nuclear diameters were measured in the junctional, labyrinth, and chorionic plate regions. Regional ESD differed significantly only in the chorionic plate between the RUPP and control groups (*P* = 0.033) (Fig. 4, G and H). The correlation between mean nuclear diameter and mean ESD yielded *R*^2^ = 0.58 (*P* = 3.8 × 10^−6^). These studies demonstrated that quantitative US can capture microstructural alterations in the PE placenta.

UFD is a powerful emerging technique capable of visualizing microvascular perfusion with high sensitivity. Osmanski *et al.* (*56*) applied UFD to a rabbit model with a human-like hemochorial placenta, where maternal and fetal circulations are highly interlaced and difficult to separate. They demonstrated that UFD is substantially more sensitive than conventional PD, successfully mapping microvessels as small as 100 μm. Beyond structural visualization, they were able to separate the pulsatile fetal flow from nonpulsatile maternal flow by analyzing the pixel-wise time variance of the Doppler central frequency. The accuracy of this discrimination was further validated using a supra-renal aortic clamp, which selectively occludes maternal flow. This functional discrimination capability provides a powerful preclinical tool for studying complex pregnancy disorders, such as FGR, which require targeted assessment of maternal versus fetal perfusion deficits.

### Clinical studies

#### Preeclampsia

The gold standard for diagnosing PE is the presence of maternal hypertension after 20 weeks of gestation, accompanied by either proteinuria or maternal organ damage. Although these diagnostic criteria are well established, UtA Doppler is additionally used for diagnostic evaluation and prognostic prediction, as abnormal placental implantation is recognized as the primary cause of PE. In PE, incomplete trophoblastic invasion of the maternal spiral arteries increases vascular resistance, leading to elevated PI and RI values as well as persistence of a diastolic notch. The PI, which reflects both systolic and diastolic flow, serves as a primary diagnostic indicator due to its sensitivity, stability, and reproducibility.

Kuc *et al.* (*75*) conducted a systematic review of 35 studies (138,571 participants, including 3654 PE cases) to identify reliable indicators for the early diagnosis of PE in first-trimester pregnancies. In this review, UtA Doppler indices (PI and RI) demonstrated a detection rate (DR; equivalent to sensitivity) of 29 to 83% at a 10% false-positive rate for predicting early-onset PE. For late-onset PE, occurring after 34 weeks, the corresponding DR ranged from 5 to 62%. These findings suggest that Doppler indices may serve as useful tools for the early diagnosis of PE. In addition, predictive models that integrate multiple categories of indicators—such as serum biomarkers and platelet markers—demonstrate greater promise than those relying solely on UtA Doppler indices (*76*).

For direct assessment of placenta perfusion, 3D power Doppler US has been investigated. Neto *et al.* (*77*) applied 3D power Doppler US to assess placental vascularization, flow, and combined vascularization-flow indices in 92 singleton pregnancies (including 8 PE cases) during the first trimester (11 to 14 weeks) and second trimester (16 to 20 weeks), confirming significantly lower values of these indices in PE cases during the second trimester. Notably, the combined vascularization-flow index demonstrated high diagnostic performance (AUC = 0.96).

#### Fetal growth restriction

FGR is a condition in which the fetus fails to achieve its genetically determined growth potential in utero due to pathological factors, representing ~30% of cases classified as small for gestational age (SGA). FGR and SGA are primarily screened using fetal biometry obtained from cross-sectional B-mode US. Estimated fetal weight (EFW) is derived from measurements of biparietal diameter, head circumference, abdominal circumference (AC), and femur length and subsequently compared with standardized growth curves. FGR or SGA is suspected when both EFW and AC fall below the 10th percentile, and FGR can be diagnosed when both measurements are below the 3rd percentile (*78*).

**Fig. 5.**
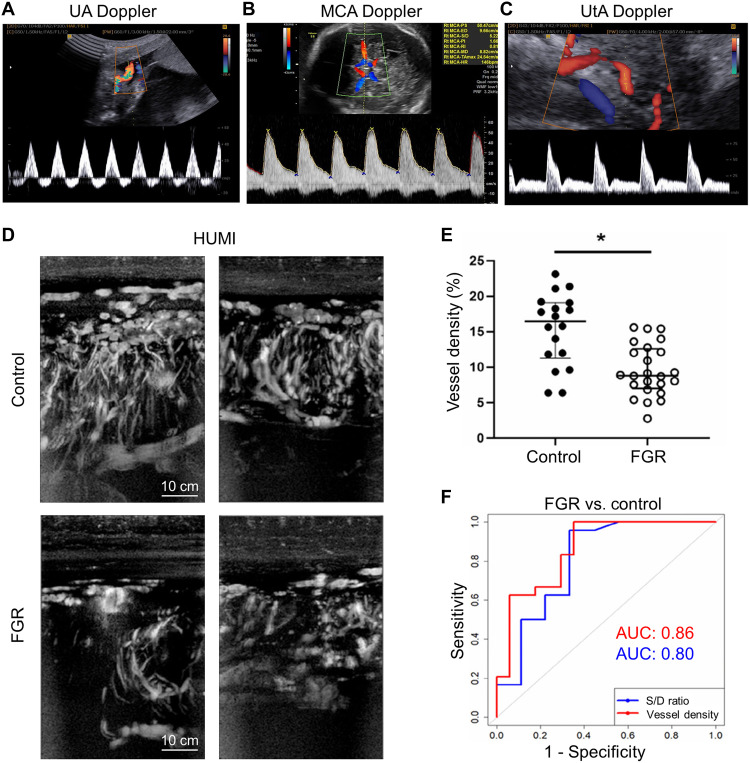
US imaging in clinical FGR studies. Doppler US examples of (**A**) the UA, (**B**) the MCA, and (**C**) the UtA. (**D**) Representative high-sensitivity US microvessel imaging (HUMI) images of control and FGR cases. (**E**) Vessel density quantification. (**F**) ROC analysis for classifying FGR and control cases using the HUMI vessel density (VD) and S/D ratio. Significance was determined by Kruskal-Wallis test, and data are presented as medians with ranges; *P < 0.05. Figures are adapted with permission from (*85*, *150*).

The primary pathophysiology of FGR involves reduced placental perfusion resulting from defective placentation, and Doppler US is widely applied to differentiate FGR from SGA (*79*). In particular, UA Doppler reflects fetoplacental perfusion resistance, making it an essential tool for predicting and managing FGR (Fig. 5A). Absent end-diastolic flow (AEDF) in UA Doppler can serve as a standalone criterion for diagnosing FGR (*78*). Unlike early-onset FGR, which occurs before 32 weeks, late-onset FGR generally exhibits less severe pathophysiology and presents with normal UA Doppler findings, prompting the recommendation of cerebral Doppler as the primary diagnostic parameter (*80*). Middle cerebral artery (MCA) Doppler is associated with the brain-sparing effect (Fig. 5B), a compensatory response to hypoxia, while the cerebroplacental ratio (CPR), defined as MCA-PI/UA-PI, is an important prognostic marker in late-onset FGR. The UtA Doppler may assist in early diagnosis, as persistent notching and elevated PI indicate an increased risk of FGR development (Fig. 5C) (*81*).

In a multicenter prospective observational study, Unterscheider *et al.* (*82*) identified the US findings strongly associated with perinatal morbidity and mortality among 1116 singleton pregnancies (24 to 36 weeks) with an EFW below the 10th percentile. Of the 1116 cases, 312 (28%) required neonatal intensive care unit admission (NICU) admission, 58 (5.2%) experienced adverse perinatal outcomes, and 8 (0.7%) resulted in perinatal death. Abnormal UA Doppler findings—AEDF, reversed end-diastolic flow, or PI ≥ 95th percentile—were significantly associated with all adverse outcomes, but their use alone remains limited for differentiating FGR from SGA. In the systematic review conducted by Alfirevic *et al.* (*83*), while UA Doppler may reduce perinatal mortality and lower the rates of cesarean delivery and labor induction in high-risk pregnancies, the supporting evidence is of moderate to low quality. Efforts are underway to enhance diagnostic performance through the integrated use of multiple Doppler indices, fetal biometry, and maternal serum markers (sFlt-1 and PAPP-A).

Rizzo *et al.* (*84*) conducted a prospective cohort study of 243 singleton pregnancies with late-onset FGR to assess the predictive value of Doppler indices. Among the 243 cases, 32.5% experienced a composite adverse perinatal outcome (CAPO), which included emergency cesarean delivery for fetal distress and NICU. In CAPO cases, UA PI was elevated, while MCA PI, CPR, and umbilical vein blood flow normalized for fetal abdominal circumference (UVBF/AC) were reduced. In multivariate logistic regression analysis, UtA PI, CPR, and UVBF/AC were independently associated with CAPO, with corresponding ROC curve areas of 0.59, 0.63, and 0.72, respectively. A multiparametric model combining these three indicators yielded an AUC of 0.75. This study suggested that Doppler findings may contribute to prognostic prediction in late-onset FGR; nevertheless, challenges regarding measurement standardization and reproducibility remain.

Lok *et al.* (*85*) used power Doppler–based high-sensitivity US microvessel imaging (HUMI) with step-by-step scanning to generate pseudo-3D placental images in FGR pregnancies (Fig. 5D). Results from 42 pregnant women (24 FGR, 18 controls) at a mean gestational age of 35 + 5 weeks were analyzed. Placental vessel density (VD) was significantly reduced in FGR cases compared with controls (9.6% versus 15.3%, *P* = 0.0004) (Fig. 5E). VD demonstrated diagnostic performance comparable to that of the UA S/D ratio (Fig. 5F). Although this study had several limitations, the hypovascularization characteristic of the FGR placenta may contribute to understanding FGR pathophysiology, risk stratification, and personalized management.

#### Placenta accreta spectrum

PAS leads to structural abnormalities at the uteroplacental interface and subsequent impaired blood flow arising from aberrant neovascularization and remodeling. B-mode US imaging enables the detection of structural abnormalities, including myometrial thinning, loss of the retroplacental clear zone, placental bulge, exophytic mass, and placental lacunae. Color and power Doppler US facilitate visualization of abnormal hemodynamics, such as turbulent or high-velocity flow within lacunae, bridging vessels, and subplacental, uterovesical, or intraplacental hypervascularity.

US imaging exhibits high diagnostic performance in the detection of PAS. A systematic review and meta-analysis by Maged *et al.* (*86*) evaluated 54 studies. Across 50 studies using 2D US, 2025 of 5406 patients were diagnosed with PAS. The pooled diagnostic performance demonstrated a sensitivity of 87%, a specificity of 86%, a positive likelihood ratio of 4.99, a negative likelihood ratio of 0.16, and an odds ratio of 34.2. Studies using 3D US were also assessed, demonstrating good diagnostic performance with a sensitivity of 73% and a specificity of 97%. This meta-analysis also suggested that diagnostic accuracy improves with the combined use of multiple markers.

**Fig. 6.**
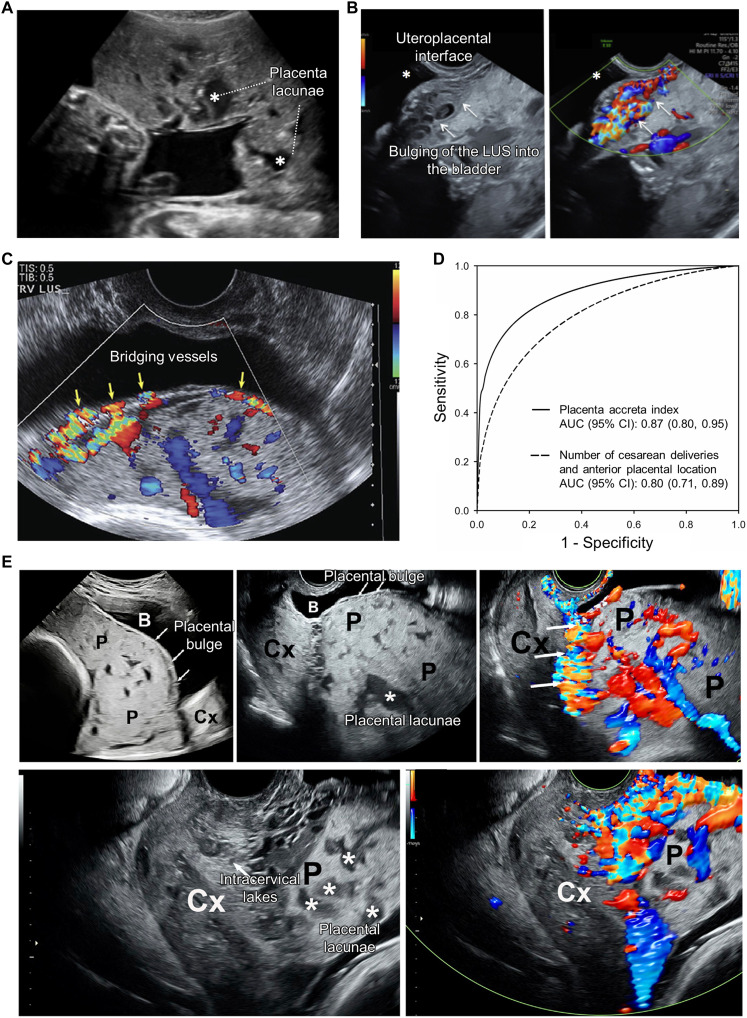
US imaging in clinical PAS studies. (**A**) B-mode image of placental lacunae. (**B**) Color Doppler image of the uteroplacental interface in PAS. LUS, lower uterine segment. (**C**) Color Doppler image of bridging vessels. (**D**) Placenta accreta index (PAI). 95% CI, 95% confidence interval. (**E**) Longitudinal B-mode and color Doppler images of the LUS and cervix (Cx) at 35 weeks of gestation in a patient with PAS. P, placenta previa. Figures are adapted from (*87*, *88*) (reused with permission) and (*90*) [CC BY (https://creativecommons.org/licenses/by/4.0/)].

Although similar US findings have been reported, diagnostic criteria, definitions of sonographic features, and examination approaches for PAS vary across studies. Accordingly, standardization of prenatal US has been strongly emphasized. The consensus on US markers and diagnostic criteria for pregnancies at risk of PAS, led by the Society for Maternal-Fetal Medicine, outlined key markers and recommended trimester-specific diagnostic approaches (*87*). In the first trimester, important markers include cesarean scar pregnancy and low implantation, whereas in the late first trimester, findings such as placental lacunae, abnormal bladder interface, uterovesical hypervascularity, and loss of the retroplacental clear zone are commonly observed (Fig. 6, A and B). Transvaginal US together with color Doppler imaging was recommended as diagnostic modality. In the second and third trimesters, key US markers include placental lacunae, abnormal uteroplacental interface, abnormal uterine contour (placental bulge), and exophytic mass. Transabdominal US was recommended as the baseline modality for confirming placental location, with transvaginal US performed as needed, and the use of color Doppler was also recommended. In the first trimester, assessment focuses on confirming the implantation site and detecting early blood flow abnormalities, whereas in the second and third trimesters, emphasis shifts to evaluating structural abnormalities and abnormal vascular flow.

Attempts to develop a quantitative scoring system for PAS diagnosis have been ongoing. A single-center retrospective study combined US indicators to propose a Placenta Accreta Index (PAI) for quantifying the risk of placental invasion (*88*). Among 184 patients with at least one prior cesarean section history who were diagnosed with placenta previa or low-lying placenta on third-trimester US, placental invasion was histologically confirmed in 54 (29%). The optimal combination for the PAI included placental location, number of prior cesarean sections, sagittal myometrial thickness, lacunae, and bridging vessels (Fig. 6C). On the basis of this combination, the PAI, a 0- to 9-point scoring system derived from logistic regression, demonstrated 72% sensitivity at a cutoff >4 points and 100% specificity at a cutoff >6 points, achieving an AUC of 0.87 for histologically confirmed PAS (Fig. 6D). This study was subsequently replicated in a contemporary clinical cohort (*89*).

Transvaginal US is being increasingly used; however, research remains limited. A retrospective analysis of prospective data by Jauniaux *et al.* (*90*) evaluated the contribution of transvaginal US among high-risk pregnant women with a history of cesarean delivery and third-trimester placenta previa or low-lying placenta. Of 111 women, 76 were diagnosed with PAS (11 accreta, 65 increta), and transabdominal and transvaginal US showed comparable prediction accuracy at 95 and 96%, respectively. Among the 72 patients undergoing hysterectomies, only a lacunae score ≥ 3 on transabdominal US showed a significant association with PAS. In contrast, transvaginal US additionally demonstrated significant associations with lower uterine segment (LUS) thickness of <1 mm, abnormal cervical structure with increased vascularity, and intracervical lakes (Fig. 6E). Transvaginal US provides more direct visualization of the LUS and cervix, enabling assessment of findings difficult to detect transabdominally. Transvaginal US may play a pivotal role in the early diagnosis of PAS during the first trimester, facilitating the detection of low-implantation pregnancy or cesarean scar pregnancy (*91*).

#### Gestational diabetes mellitus

The oral glucose tolerance test is the gold standard for diagnosing GDM. US imaging serves as an adjunctive modality, primarily used to assess fetal and placental complications associated with GDM. B-mode US is applied to evaluate macrosomia and adipose tissue deposition through measurements of AC, EFW, and liver size and to confirm placental hypertrophy by assessing increases in placental thickness and volume. Doppler US evaluates reduced blood flow resistance in GDM through UtA, UA, and placental bed flow indices; assesses vascularization using 3D power Doppler; and monitors fetal blood flow compensation through MCA Doppler. Placental fibrosis and increased stiffness in GDM can also be detected using SWE.

In a randomized controlled trial, Kjos *et al.* (*92*) investigated the inclusion of B-mode US–based AC measurement in the insulin prescription criteria for 98 pregnant women with GDM and fasting plasma glucose (FPG) levels between 105 and 120 mg/dl. The standard group (*n* = 49) received insulin therapy universally, whereas the experimental group (*n* = 49) underwent monthly fetal AC measurements and received insulin therapy only when AC was ≥70th percentile or FPG was ≥120 mg/dl. Macrosomia rates and neonatal complication risks showed no significant difference between the two groups. Although the cesarean delivery rate was higher in the experimental group, 38% of participants were safely managed without insulin therapy. These findings suggest that integrating US-based fetal growth assessment into insulin administration decisions could help reduce unnecessary insulin use.

Fatihoglu *et al.* (*93*) investigated hemodynamic changes using UA and MCA Doppler during early gestation (18 to 22 weeks, second trimester) in 60 women with GDM and 61 healthy controls, assessing their potential as early warning indicators of GDM. Using a 3.5-MHz convex US probe, peak systolic velocity (PSV), EDV, PI, RI, and S/D ratio were measured in both UA and MCA Doppler. MCA Doppler demonstrated significantly lower PSV in the GDM group (28 cm/s versus 32 cm/s, *P* = 0.037), with PSV < 35.5 cm/s yielding a predictive sensitivity of 41% and a specificity of 78.3% for GDM. In contrast, UA Doppler parameters showed no significant differences between the groups. This study suggested that MCA Doppler may detect fetal hemodynamic alterations in the earlier stages of gestation.

Lai *et al.* (*94*) established reference ranges for placental Young’s modulus in normal pregnancies (223 of 259 women) using 2D SWE and confirmed significantly increased stiffness in 36 GDM cases (22 diet-controlled A1GDM and 14 medication-requiring A2GDM). Medication-requiring GDM showed higher Young’s modulus than diet-controlled GDM, indicating placental changes linked to more severe metabolic abnormalities and suggesting a potential diagnostic role for 2D SWE. Çelik *et al.* (*95*) used superb microvascular imaging and SWE to assess placental vascularity and elasticity. Compared with 20 normal pregnancies, the 20 women with GDM showed significantly higher values in both parameters. While these findings are promising, large-scale studies with pathological validation are warranted.

### Technical challenges and solutions

US imaging relies on the characteristics of acoustic signals, and its performance varies with tissue depth, acoustic path, and equipment settings. These physical factors, together with dependence on instrumentation and operator technique, impose several inherent limitations in placental imaging.

First, US imaging has acoustic limitations. In cases of a posterior placenta or high maternal body mass index (BMI), acoustic attenuation hampers visualization of placental structures. Furthermore, a trade-off exists between resolution and penetration depth: A high center frequency provides high resolution with shallow penetration, whereas a low center frequency yields deeper penetration at the expense of resolution. Among US techniques, pulse-compression methods (e.g., coded excitation) provide effective solutions to this limitation (*96*). By increasing the time-bandwidth product, these methods preserve resolution while allowing greater transmit energy, enhancing signal-to-noise ratio (SNR) and extending effective imaging depth.

Second, the quality of US imaging is affected by fetal movement, uterine contractions, maternal respiration, and organ motion. These factors may lead to continuous changes in placental position and shape, producing motion artifacts in US imaging and reducing measurement reliability. Motion correction techniques applied in conventional US of other organs or in modalities such as MRI and CT could prove beneficial (*97*).

Third, US imaging lacks standardized approaches to measurement and interpretation. US imaging is highly influenced by equipment settings (e.g., TGC), with signal processing differing across devices and strong operator dependence arising from probe handling, position, and angle. In addition, variability in the diagnostic and prognostic criteria for severity across perinatal diseases further contributes to the lack of standardization. Artificial intelligence (AI)–driven US techniques designed to minimize variability may offer substantial benefits (*98*).

Fourth, current B-mode and Doppler US techniques have limitations in directly evaluating placental circulation, which is associated with exchange efficiency between the intervillous spaces and villous capillaries, where oxygen and nutrient transfer takes place. Evaluation of this circulation is essential for predicting fetal growth and managing perinatal outcomes. 3D power Doppler, which can visualize and quantify the entire placental vascular network, has recently benefited from advances in UFD techniques that have enhanced vascular detection sensitivity (*99*). This improvement enables visualization at the microvascular level and allows assessment of 3D blood flow distribution and perfusion based on blood flow volume, facilitating placental circulation evaluation.

## PHOTOACOUSTIC TECHNIQUES FOR PLACENTA IMAGING

### Preclinical studies

PA imaging is rapidly emerging as a powerful tool in preclinical studies of the placenta and fetus, primarily due to its ability to provide simultaneous acquisition of vascular structure, sO_2_, and molecular information. This is especially critical during the middle to late stages of pregnancy when rapid fetal growth leads to increased oxygen demand (*100*). Early detection of placental dysfunction through PA imaging can provide essential guidance for timely interventions such as improving maternal oxygenation, administering appropriate therapeutics, or adjusting the timing of delivery to safeguard fetal health (*101*). Moreover, direct assessment of fetal oxygenation, not only for placental function, is critical for preventing severe perinatal complications such as hypoxic-ischemic encephalopathy and cerebral palsy. However, current standard fetal monitoring techniques provide only indirect information, limiting the direct measurement of oxygenation status (*102*). In practice, various PA imaging techniques have been used for real-time analysis and longitudinal monitoring of whole-body or specific regions of interest in small animals (*103*). Extending these approaches to the placenta and fetus has been reported to enable effective acquisition of multiparametric information tailored to experimental requirements (*103*, *104*).

**Fig. 7.**
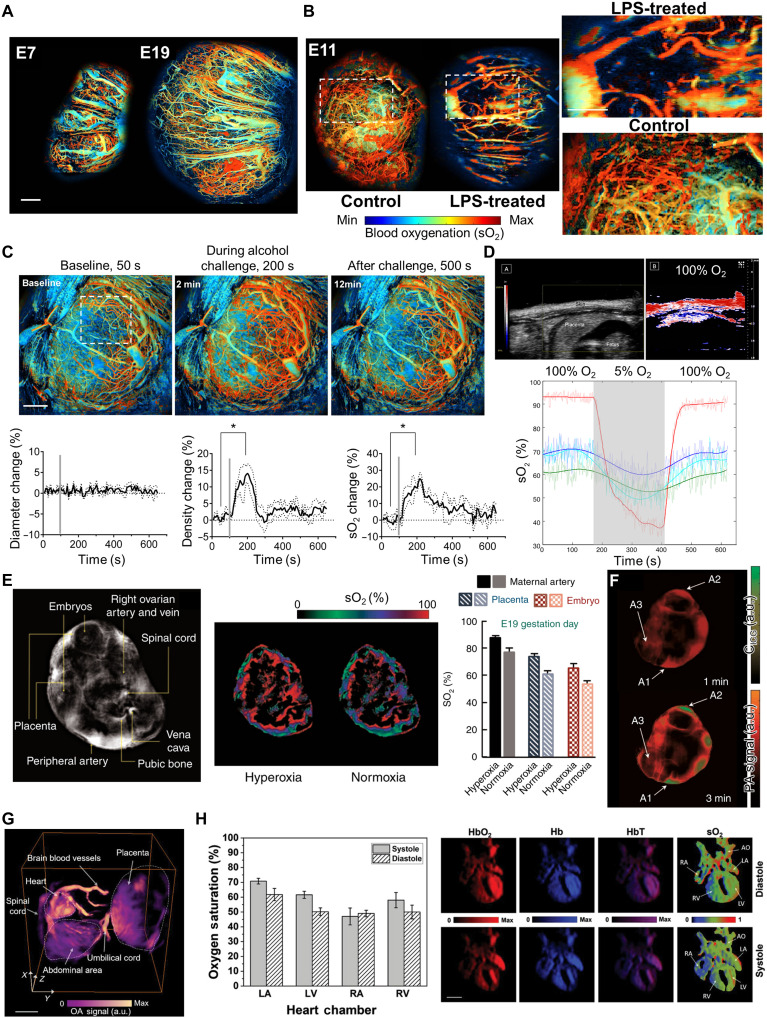
Preclinical placental studies with PA imaging. (**A**) UFF-PAM images of the sO_2_ mapping of a mouse placenta at E7 and E19. (**B**) UFF-PAM images of placentas of the LPS-treated mouse and control mouse at E11. (**C**) Placental hemodynamic responses monitored by UFF-PAM. (**D**) B-mode US image and PA-based sO_2_ image of placenta and sO_2_ variation during the sequence of hyperoxygenation, hypoxia, and hyperoxygenation from skin (red), mesometrial triangle (light blue), labyrinth zone (dark blue), and basal zone (green). (**E**) In vivo tomographic scan of E19 pregnant mouse and sO_2_ distributions under hyperoxic and normoxic conditions. (**F**) Spectrally unmixed indocyanine green (ICG) distribution in placental region. (**G**) 3D image of an embryo at GD16.5 acquired with volumetric optoacoustic spectroscopy. (**H**) sO_2_ distribution in embryonic heart chambers in the systolic and diastolic phases. a.u., arbitrary units; OA, optoacoustic; RA, right atrium; LA, left atrium; RV, right ventricle; LV, left ventricle; AO, aorta. The images are adapted from (*103*) [CC BY-NC (https://creativecommons.org/licenses/by-nc/4.0/)], (*104*, *105*, *108*) [CC BY (https://creativecommons.org/licenses/by/4.0/)].

Zhu *et al.* (*103*) developed ultrafast functional PAM (UFF-PAM), combined with an intravital window, which enables high-speed quantitative monitoring of placental vascular structure and sO_2_ dynamics in mice, with a lateral spatial resolution of ~10 μm. While fetuses in the window-implanted group exhibited ~33.8% lower body weight compared to controls, histological analysis revealed no evident morphological or physiological abnormalities. They longitudinally monitored placental vascular development from embryonic day 7 (ED7) to ED19 (Fig. 7A), quantifying changes in vessel diameter, VD, and the spatiotemporal distribution of sO_2_. The study further monitored a lipopolysaccharide (LPS)–induced inflammation model up to ED13, revealing reduced vessel diameter and density compared to controls, indicating impaired microvascular development (Fig. 7B). Placental sO_2_ was higher relative to controls, which the authors attributed to a disruption of the normal low-oxygen environment essential for healthy placental development. In addition, leveraging the high imaging speed, the system enabled real-time monitoring of acute responses such as cardiac arrest induced by oxygen/nitrogen modulation and maternal alcohol exposure, capturing dynamic changes in placental vessel diameter, density, and sO_2_ over several minutes (Fig. 7C). Arthuis *et al.* (*105*) used combined B-mode US and overlapped PA imaging in a pregnant rat model to quantitatively assess placental sO_2_ changes during transitions from hypoxia to hyperoxia. They compared the maternal skin, the labyrinth, the basal zone, and the mesometrial triangle region in the placenta. The sO_2_ in the skin dropped markedly (~70%), whereas the placental regions showed only a modest decrease (~10 to 20%) and rapidly recovered under hyperoxic conditions (Fig. 7D). These findings highlight that the placenta plays a protective role by maintaining a relatively stable level of oxygenation even under maternal hypoxia.

Beyond placental imaging, several studies have extended PA approaches to directly capturing fetal and embryo images. Laufer *et al.* (*106*) used a Fabry-Pérot (F-P) US sensor–based PAT system to visualize murine embryos both in vivo and ex vivo, successfully identifying major anatomical structures such as the heart, head, spinal region, liver, and other embryonic vascular systems at depths up to 10 mm. Not only for structural imaging, PA also enables multiparametric assessment by capturing spatiotemporal changes in sO_2_ and analyzing the distribution of exogenous contrast agents through its molecular sensitivity. Menozzi *et al.* (*107*) demonstrated that 3D diffractive acoustic tomography (3D-DAT) using single slit for near-isotropic, high-speed, large-FOV imaging, and among the 3D-DAT approaches, they used diffractive PAT (DPAT) to noninvasively monitor embryonic blood sO_2_ in pregnant BALB/c mice throughout gestation (E8.5 to E18.5). Specifically, mice exposed to per- and polyfluoroalkyl substances (PFAS) served as the disease group to assess the effects of PFAS exposure on embryonic oxygen metabolism and development. Using high-frequency US for comparison, they showed that functional changes precede structural alterations. DPAT confirmed normal fetal oxygenation dynamics, with embryonic vascular sO_2_ remaining ~21% lower than maternal levels during E12 to E15, whereas PFAS exposure significantly increased the embryonic-to-maternal vessel sO_2_ ratio. They validated increased oxidative stress in the fetal brain of the PFAS group using oxidative stress markers, demonstrating that PFAS exposure induces abnormal hyperoxia and elevates brain oxidative stress. Basak *et al.* (*108*) used a ring-shaped optoacoustic tomography system to achieve real-time separation and tracking of maternal respiration (~0.7 Hz) and embryonic heartbeats (~4 to 5 Hz) in pregnant mice. They performed oxygen challenge experiments and quantified cross-sectional images to assess changes in sO_2_ across maternal, placental, and embryonic regions under normoxic and hyperoxic conditions. The magnitude of sO_2_ variation was greatest in maternal peripheral arteries, followed by the placenta, and smallest in the embryo (Fig. 7E). In addition, tail vein injection of indocyanine green (ICG) allowed dynamic monitoring of tracer kinetics. Strong signal enhancement was observed in maternal arteries and placental regions, whereas no signal increase was detected in the embryo, thereby enabling observation of the placental barrier (Fig. 7F). Hatami *et al.* (*104*) used a volumetric optoacoustic spectroscopy to noninvasively track murine embryonic cardiac dynamics with high spatial (~100 μm) and temporal (~10 ms) resolution. The study visualized entire embryonic structures in vivo from GD14.4 to 17.5, successfully distinguishing key anatomical structures including the heart, brain vessels, and spinal cord (Fig. 7G). They also spatially identified the growth of atria and ventricles during embryonic development and captured their dynamic changes across systole and diastole in real time. Multiwavelength imaging enabled mapping of sO_2_ within each cardiac chamber, revealing cycle-dependent variations in fetal circulation (Fig. 7H). Several studies applied exogenous contrast agents to explore molecular transport across the placenta and fetus. Zhang *et al.* (*109*) administered poly[(9,9-dioctylfluorenyl-2,7-diyl)-co-(1,4-benzo-{2,1′,3}-thiadiazole)] and poly[2-methoxy-5-(2-ethylhexyloxy)-1,4-phenylenevinylene] polymer dots to pregnant mice, demonstrating their preferential accumulation in the placenta while no detectable signal was observed within the fetus, thereby confirming the function of the placental barrier. Moses *et al.* (*110*) demonstrated that silicon naphthalocyanine encapsulated within a biocompatible polymeric poly(ethylene glycol)-block-poly(ε-caprolactone) methyl ether nanoparticles preferentially accumulated in the murine placenta, highlighting the potential of this approach for the diagnosis of ectopic pregnancy. Moreover, the nanoparticles remained restricted by the placental barrier, ensuring no fetal exposure, while NIR irradiation induced sufficient localized heat (>43°C) to disrupt placental function, enabling selective photohyperthermia of the targeted embryo. This demonstrated the potential of the approach as a theranostic modality capable of both diagnosing and treating ectopic pregnancy.

**Fig. 8.**
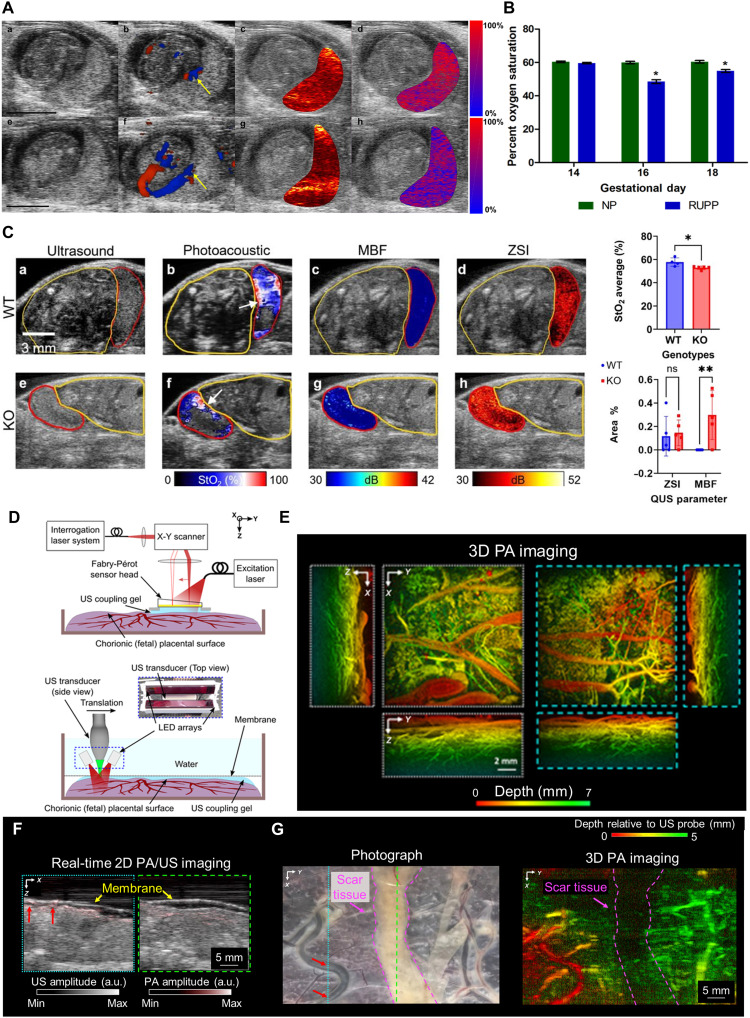
Preclinical placental disease model studies and clinical translational studies using PA imaging. (**A**) B-mode US images, color Doppler US overlapped, PA signal intensity overlapped, and sO_2_ overlapped images of the placental environment of RUPP (top row) and normal pregnant (NP; bottom row). (**B**) Comparison of average placental sO_2_ between RUPP and NP groups. (**C**) US, PA, and QUS maps from placentas in the WT and Slc20a2 genetic KO groups. Quantitative comparison of WT and KO groups. QUS, quantitative US spectral; MBF, mid-band fit; ZSI, zero-slope intercept; ns, not significant. (**D**) F-P–based planar sensor PA imaging system (top) and clinical linear array US probe–based system (bottom). (**E**) PA images of the chorionic placental vasculature of a normal term placenta using F-P–based system. (**F**) PA and US imaging of the chorionic placental vasculature in an untreated part using clinical linear array–based system. (**G**) Photograph and PA image of the scar tissue from photocoagulation treatment. The images are adapted from (*101*, *118*) [CC BY (https://creativecommons.org/licenses/by/4.0/)] and (*114*) [CC BY-NC-ND (https://creativecommons.org/licenses/by-nc-nd/4.0/)].

Several studies have quantitatively and comprehensively captured physiological changes in the placenta and fetus across various obstetric disease models. Shan *et al.* (*111*) used a 256-element cylindrically focused array-based PAT system to investigate hemodynamic alterations in the fetal brain following acute prenatal ethanol exposure. They administered 20% ethanol (3 g/kg) to GD17 pregnant mice and imaged the fetal brain, tracking changes in vessel diameter, vascular density, and sO_2_. Within 40 min of ethanol administration, vessel diameter decreased by ~31%, vascular density by 25%, and fetal vascular sO_2_ by about 40%. Lawrence *et al.* (*101*, *112*) applied photoacoustic ultrasound (PAUS) imaging to a RUPP rat model, analyzing both structural information along with sO_2_ measurement (Fig. 8A). As a result, the placental sO_2_ levels in the RUPP group were ~10% lower compared to the control group (Fig. 8B). These results were validated by elevated mean arterial pressure measured using a saline-filled catheter, increased protein excretion, and hypoxia-inducible factor-1α staining. Yamaleyeva *et al.* observed fetal growth and placental sO_2_ in groups of mice with hypertension induced by L-NAME administration and angiotensin-converting enzyme 2 knockout (KO) mice, in which chronic perfusion deficits led to placental hypoxia (*113*). In particular, the study used multispectral PA overlapped with US to distinguish detailed placental regions such as the labyrinth (L) and the junctional zone plus decidua (JZ + D) in normal mice and identified changes in sO_2_ within these regions as maternal inspired oxygen levels were altered from 100 to 20%. Edwards *et al.* (*114*) induced placental dysfunction using a Slc20a2 genetic KO model and used quantitative US and PA imaging to jointly analyze quantitative US spectral (QUS) parameters and sO_2_. PA imaging revealed that the KO group exhibited lower mean placental sO_2_ compared to the wild type (WT). In addition, QUS analysis showed that both mid-band fit and zero-slope intercept, which are parameters reflecting the size, concentration of tissue scatterers, and acoustic impedance, were significantly elevated in the KO group, indicating structural abnormalities such as calcification (Fig. 8C). Noble *et al.* (*115*) used PA imaging in Sprague Dawley rat fed with an iron-restricted diet during pregnancy to evaluate the effects of iron-deficient anemia on placental and fetal sO_2_. sO_2_ was measured under normoxic, hypoxic, and hyperoxic conditions, and in the anemia group, FGR and an overall reduction in hemoglobin levels (measured by the HemoCue 201+ system) were observed. However, PA-based measurements revealed no major differences in placental and fetal oxygenation compared with controls, except for localized increases in sO_2_ in particular regions such as the fetal face under hyperoxic conditions. The authors discussed that this may reflect fetal and placental adaptations to ensure fetal survival in a suboptimal gestational environment. In particular, these three studies commonly demonstrated that the PAUS multimodal strategy complements structural and functional information, overcomes existing limitations, and maximizes the advantages of PA imaging.

### Technological challenges and solutions toward clinical translation

Clinical applications of PA imaging for the placenta and fetus are still in their nascent stages, and in vivo studies in living pregnant women have been limited due to several practical challenges. The deep location of the placenta within the maternal abdomen—often 3 to 5 cm beneath layers of subcutaneous fat, abdominal wall, and uterine muscle—significantly restricts light penetration because of strong optical scattering and absorption. Given that PAM generally achieves only a few millimeters and PACT typically provides penetration of only several centimeters even under favorable NIR conditions, these physical constraints hinder the broader application of PA-based placental imaging across preclinical and clinical studies (*116*). These limitations are even more restrictive for PAM, which is often used for higher-resolution assessments; it can effectively operate only within confined to exposed tissue, early-gestation mouse models, or abdominal window preparations. Consequently, current PA studies related to placental assessment have largely remained exploratory, relying primarily on ex vivo human placentas obtained after delivery or small-animal models under controlled or surgically facilitated imaging conditions. In this section, we summarize exploratory clinical studies that investigate the translational potential of PA-based placental assessment, as demonstrated in preclinical research, and highlight approaches that may facilitate further scalability and clinical translation of PA imaging.

Xia *et al.* (*117*) delivered light directly to the placental surface using an optical fiber and used a fiber-optic hydrophone attached to the fiber for US tracking of its position, enabling PA signal acquisition with a clinical linear US probe to visualize the placental surface and superficial vasculature. The study confirmed that the wavelength-dependent PA signal intensities matched the absorption spectrum of deoxygenated hemoglobin, thereby validating the feasibility of visualizing vascular oxygenation and demonstrating further potential for real-time coregistration of PA and US images using ultrasonic tracking. Maneas *et al.* (*118*) investigated human placentas ex vivo after delivery, including cases with twin-to-twin transfusion syndrome (TTTS), and visualized the chorionic surface and subsurface vasculature up to a depth of ~7 mm. The study used two systems: an F-P planar sensor–based PA system and a light-emitting diode (LED) array combined with a clinical linear array–based PAUS system (Fig. 8D). The F-P system provided visualization of microvascular structures (Fig. 8E), whereas the PAUS system enabled rapid 2D imaging along with simultaneous US structural information, showing their complementary advantages (Fig. 8F). They compared a normal term placenta with a placenta from TTTS pregnancies treated with laser photocoagulation and identified ablation sites within the treated placenta (Fig. 8G). Conventional US imaging and white-light fetoscope often fail to detect abnormal deep vascular anastomoses in the placenta, leading to incomplete treatment; however, this study demonstrated the clinical potential of PA imaging as a minimally invasive guidance tool for fetal therapy by enabling 3D visualization of the human placental vasculature.

These studies have demonstrated the potential clinical utility of PA imaging for the human placenta. Nevertheless, substantial technical challenges remain for translation to in vivo applications in pregnant women. One of the main challenges is the restricted optical penetration depth caused by strong scattering and absorption within biological tissues. The light must traverse ~23 mm of abdominal tissue, including subcutaneous fat and muscle, to reach the placenta, and accessibility is further reduced when the placenta is attached to the posterior uterine wall, where it is located deeper near the maternal spine (*119*). To address these depth limitations, strategies for optimizing light delivery have been investigated, and Huda *et al.* (*116*) proposed an approach based on light transport simulations that could enhance imaging depth by ~1.6 times. They conducted Monte Carlo light transport simulations using a multilayered model and identified that increasing the beam area was the most effective strategy to enhance fluence at greater depths, which was subsequently implemented through geometrically optimized light delivery. Another approach to optimizing the light delivery to deeper tissue is multiangle illumination, in which optimizing the arrangement and incidence angle of optical fibers, rather than relying on fixed-angle illumination, can improve quality by enhancing the SNR and contrast-to-noise ratio (CNR) at greater imaging depths (*120*). Last, unlike the conventional transabdominal approach, an endocavity PA probe introduced via the cervix can substantially shorten the light delivery path, thereby avoiding the depth issues (*121*). Another approach to optimizing light delivery is multiangle illumination, where adjusting the arrangement and incidence angle of optical fibers can improve SNR and CNR at greater depths, albeit with added system complexity and cost (*120*). Recently, various deep learning–based strategies have been proposed to compensate for the low SNR inherent to low-dosage light sources such as LEDs (*122*). Last, unlike the conventional transabdominal approach, a transvaginal PA probe introduced via the cervix can substantially shorten the light delivery path, thereby avoiding the depth issues (*121*).

One of the other major constraints in the clinical application is the potential for laser-induced damage to fetal tissues, particularly the eyes and skin, since visible to NIR (400 to 1400 nm) wavelengths commonly used in PA imaging are classified as the retinal hazard region (*123*). Although fetal skin contains less melanin, potentially reducing thermal risk, strict compliance with the maximum permissible exposure standards for skin at different wavelengths is required, as established by the American National Standards Institute (*124*). Beyond these regulatory limits, a more comprehensive consideration of laser safety is crucial for any technology with translational intent, including fetal sensitivity to optical and thermal exposure and potential tissue temperature rise under repeated pulsed illumination (*125*). In this context, as a strategy to minimize the risks associated with laser exposure, it is noteworthy that the molecular specificity of PA imaging can be leveraged for indirect monitoring of the placenta and fetus. For example, one of the key indicators of fetal distress is meconium aspiration syndrome, which is observed as meconium staining of the amniotic fluid when meconium is released into it, and multispectral PA imaging can selectively detect it by leveraging its unique molecular absorption properties (*126*). In addition, although molecular markers of fetal health in the amniotic fluid are now accessible only through invasive procedures such as amniocentesis, PA imaging could noninvasively detect biomarkers of infection and inflammation, including IL-6 and matrix metalloproteinase-8 (*127*). This approach minimizes the risk of direct energy exposure to the fetus while providing quantitative and molecular information, offering a safe and practical pathway to maximize clinical value.

Placental imaging studies using US and PA, as covered in the “Ultrasound techniques for placenta imaging” and the “Photoacoustic techniques for placenta imaging” sections, are summarized in Table 1 according to perinatal disorder and potential application. PAS is characterized by the depth of trophoblastic invasion, making clear visualization of anatomical boundaries essential for diagnosis. Because severe cases often extend beyond the uterus into adjacent pelvic structures (e.g., the bladder or pelvic sidewall), most studies have relied on conventional B-mode and Doppler US. Advanced US techniques have been rarely applied due to limited penetration relative to the deep anatomical targets required for PAS assessment. Although emerging US technologies that improve acoustic penetration may offer value in evaluating deeply invasive PAS, further investigation is needed.

**Table 1.** Summary of placental US and PA studies. REDF, reversed end-diastolic flow; FPI, feto-placental index; PSV, peak systolic velocity; SMI, superb microvascular imaging; PHT, photohyperthermia; ZSI, zero-slope intercept; MBF, mid-band fit. For studies categorized under “Others,” the implications include potential applicability to perinatal disorders.

Perinatal disorder	Imaging	Reference	Modality	Implication (indicator, potential application)
PE	Conventional	Kuc *et al.* (*75*)	UtA Doppler US	Evaluation of UtA Doppler diagnostic performance in the first trimester of pregnancy (UtA PI↑, UtA RI↑)
	Advanced	Gleed *et al.* (*73*)	Quantitative US (HKD, VisualSonics Vevo 2100, LZ 250 probe)	Detection of microstructural alterations in the PE placenta (scatterer clustering α↓, kurtosis values↓)
		Markel *et al.* (*74*)	Quantitative US (ESD, VisualSonics Vevo 2100, LZ 250 probe)	Detection of microstructural alterations in the PE placenta (ESD↓)
		Neto *et al.* (*77*)	Doppler US (GE Voluson E8 with RAB 4-8D probe)	Feasibility of 3D power Doppler US for PE diagnosis (vascular indices↓)
		Lawrence *et al.* (*101*)	PAT (US overlapped)	Placental oxygenation (sO_2_↓)
		Yamaleyeva *et al.* (*113*)	PAT (US overlapped)	Monitoring of oxygenation changes by placental region and fetal growth (sO_2_↓)
FGR	Conventional	Unterscheider *et al.* (*82*)	B-mode US, Doppler US (GE Voluson E8)	Validation of EFW and UA Doppler predictive performance for adverse outcomes in FGR (abnormal UA Doppler; AEDF, REDF, PI↑)
		Alfirevic *et al.* (*83*)	Doppler US	Clinical utility and limitations of UA Doppler in FGR (UA PI↑, UA RI↑, UA S/D ratio↑)
		Rizzo *et al.* (*84*)	Doppler US (Samsung Medison W80, Hera W10, 1–8-MHz volumetric probe)	Assessment of the predictive value of Doppler indices for CAPO in late-onset FGR (UA PI↑, MCA PI↓, CPR↓, UVBF/AC↓)
	Advanced	Meyer *et al.* (*71*)	B-mode US, Doppler US (VisualSonics Vevo 2100)	Elucidation of FGR progression by high-frequency US (placental dimension↓, FPI↓, UA EDV↓, UA RI↑, UA S/D ratio↑)
		Rahman *et al.* (*72*)	M-mode US, Doppler US, (VisualSonics Vevo 2100, 40-MHz linear probe)	Improving the sensitivity of UA PI through UA waveform decomposition (reflection coefficient↑)
		Lok *et al.* (*85*)	3D power Doppler US (Verasonics Vantage 256, GE LOGIQ E9, GE 9L probe)	Feasibility of US microvessel imaging in FGR diagnosis and comparative performance with UA Doppler (VD↓)
PAS	Conventional	Maged *et al.* (*86*)	2D/3D US	Evaluation of the diagnostic performance of US imaging for PAS (e.g., myometrial thinning, bridging vessels, etc.)
		Shainker *et al.* (*87*)	B-mode US, Doppler US	Recommendations on trimester-specific key US markers and diagnostic approaches for PAS detection
		Rac *et al.* (*88*)	B-mode US, color Doppler US	Proposal of PAI for predicting placental invasion risk
		Happe *et al.* (*89*)	B-mode US, color Doppler US	Validation of PAI in a contemporary clinical cohort
		Jauniaux *et al.* (*90*)	B-mode US, color Doppler US	Performance evaluation of transvaginal US in PAS diagnosis and comparison with transabdominal US (e.g., placental position, vascularity, lacunae score, etc.)
GDM	Conventional	Kjos *et al.* (*92*)	B-mode US	Utility of B-mode US in GDM management
		Fatihoglu *et al.* (*93*)	B-mode US, Doppler US (Toshiba Medical Xario, 3.5-MHz convex probe)	Evaluation of Doppler US for early GDM diagnosis (MCA PSV↓)
	Advanced	Lai *et al.* (*94*)	B-mode US, 2D SWE (SuperSonic Aixplorer, 1–7-MHz linear probe)	Feasibility of 2D SWE in GDM diagnosis (Yong’s modulus↑)
		Çelik *et al.* (*95*)	B-mode US, SMI, SWE (Canon Medical Aplio 500, PVT-375BT convex probe)	Feasibility of placental vascularity and elasticity indices in GDM diagnosis (placental vascularity↑, placental elasticity↑)
Others	Advanced	Zhu *et al.* (*103*)	Ultrafast functional PAM (UFF-PAM)	Placental growth and acute response to drugs administration and respiratory conditions; vessel structure, oxygenation changes (PE, FGR)
		Arthuis *et al.* (*105*)	PAT (US overlapped)	Placental oxygenation during oxygen challenge (PE, FGR)
		Laufer *et al.* (*106*)	PAT (F-P based)	Ex vivo and in vivo fetal vascular structure (PAS, GDM)
		Menozzi *et al.* (*107*)	DPAT	Noninvasive longitudinal assessment of embryonic blood sO_2_, evaluation PFAS-related metabolic, developmental effects, identification normal embryonic sO_2_ patterns (FGR)
		Basak *et al.* (*108*)	PAT	Embryo heartbeat and oxygenation, ICG kinetics in placental region (FGR)
		Hatami *et al.* (*104*)	PAT	Fetal growth and heartbeat monitoring, fetal atrium and ventricle oxygenation (FGR)
		Zhang *et al.* (*109*)	PAT (US overlapped)	Biodistribution of exogenous contrast agents
		Moses *et al.* (*110*)	PAT (US overlapped)	Biodistribution of exogenous contrast agents and feasibility for PHT
		Shan *et al.* (*111*)	PAT	Vascular structure and oxygenation of the fetal brain (vessel diameter↓, VD↓, sO_2_↓) (PAS, GDM)
		Edwards *et al.* (*114*)	PAT (US overlapped)	Structural and functional changes combined with QUS parameters (sO_2_↓, ZSI↑, MBF↑) (PE)
		Noble *et al.* (*115*)	PAT (US overlapped)	Placental and fetal oxygenation, fetal growth monitoring (FGR)
		Xia *et al.* (*117*)	PAT	Ex-vivo human placental vascular imaging (PAS, GDM)
		Maneas *et al.* (*118*)	PAT (F-P based, US overlapped)	Observation of laser photocoagulation treatment regions in TTTS placentas

## FUTURE DIRECTIONS AND EMERGING TRENDS

### 3D/4D and hybrid imaging

The placenta is a large, structurally complex organ with morphology and function that evolve throughout pregnancy. 3D imaging enables visualization of the entire placental structure, vascular network, and villous architecture, supporting comprehensive assessment and revealing spatial heterogeneity in placental function. Acceleration of existing 3D imaging techniques (e.g., 3D power Doppler and PAT) through GPU-based computation could reduce processing time, thereby enabling time-resolved 3D imaging and the implementation of 4D imaging. 4D US imaging has been increasingly explored in certain applications, such as brain imaging (*128*, *129*). A comparable approach could be extended to the placenta, where PA imaging, with its faster acquisition time, may enable more straightforward implementation. 3D/4D imaging has potential applications not only in the diagnosis and prediction of perinatal diseases but also in elucidating fundamental pathophysiology and monitoring treatment by evaluating placental structure, blood flow, and function in a spatiotemporal context. The next step toward clinical use will be validating the safety and robustness of real-time 3D/4D imaging in large animal pregnancy models.

Furthermore, the integration of multimodal approaches into 3D/4D imaging may yield complementary information. Studies using hemispherical-array US transducers to simultaneously acquire 3D PAT and ULM at the same site have demonstrated the ability to obtain molecular and functional information through PAT, together with detailed microvascular structural information through ULM (*130*). This fusion imaging approach, capitalizing on the complementary strengths of individual modalities, is anticipated to improve both the precision and versatility of imaging analyses.

### Wearable and point-of-care devices

Placental function evolves throughout pregnancy, and perinatal disorders (e.g., PE, FGR, and PAS) often present with localized and intermittent pathophysiology during the early stages. This underscores the importance of continuous, repeated point-of-care (POC) placental monitoring, and wearable patch-type US and PA imaging devices can support such POC applications.

Recent studies have extensively investigated wearable US using bioadhesive layers, with newer approaches enabling patch-type monitoring of blood flow (*131*, *132*). Because these technologies can also measure vital signs such as cardiac function and blood pressure, their application to placental monitoring could markedly enhance POC performance (*133*). PA imaging can also be applied in wearable and POC monitoring. Recent studies have demonstrated patch-and watch-type PA devices capable of continuous vascular monitoring, suggesting that similar systems placed on the maternal abdomen could enable noninvasive, longitudinal assessment of placental hemodynamics and oxygenation (*134*, *135*). Such approaches—using backpack-style light source/DAQ units or patch-type systems incorporating vertical-cavity surface-emitting laser diodes—have enabled wearable PA imaging and demonstrated the potential for further miniaturization and practical device development through compact optical system designs. In addition, a transparent US transducer that aligns the optical and acoustic paths will enhance practicality by enabling seamless integration of PA and US (*136*–*139*). For wearable and portable platforms, translational success will hinge on technical miniaturization and early feasibility testing in pregnant women.

### Theranostics and image-guided interventions

Placenta-specific theranostic strategies are under active investigation; however, safety concerns for pregnant women and fetuses have thus far limited their application to the preclinical stage. PA offers advantages for theranostic guidance by enabling quantitative assessment of nanoparticle accumulation and optimizing treatment timing through absorbance-based molecular contrast and multispectral unmixing (*110*). US has been used to facilitate drug and gene transfer via microbubbles (*140*). Recently, nonviral strategies using US and microbubbles have demonstrated placental gene regulation (sonoporation), offering a precision-targeted delivery approach that can be performed concurrently with imaging (*141*). Complementary use of US and PA in theranostics could enable precise targeting of the placental-vascular boundary and allow immediate postprocedural assessment of therapeutic efficacy and residual lesions. However, safety and validation remain the most critical considerations, and these must be rigorously addressed in a stepwise manner before extending applications to pregnant women and fetuses.

For placenta-related surgical interventions, US is widely used owing to its safety profile. It serves as a tool for navigation and verification in procedures such as TTTS, twin reversed arterial perfusion sequence, and placental chorioangioma. High-intensity focused US has also been used to treat cesarean scar pregnancy but only in the postpregnancy setting. For therapeutic use during pregnancy, low-intensity focused US could be considered. The theranostic integration of PA imaging and targeted agents will require rigorous safety evaluation before initiation of early-phase human studies.

### AI-enhanced predictive modeling

AI is widely applied to automatic segmentation of the placental region, a process essential for functional evaluation through quantitative metrics (*142*). Unlike fetal imaging, where structural assessment predominates, functional imaging is particularly critical for the placenta. AI-based segmentation enables (i) quantification of the entire placental area, (ii) calculation of regional indicators by subdividing the placenta into detailed segments, and (iii) precise differentiation of adjacent structures such as the uterine wall, umbilical cord, and fetal boundaries. Furthermore, automated analysis enhances reproducibility, shortens processing time to facilitate rapid diagnosis, and contributes to standardization. The attention-enhanced U-Net architecture may be beneficial for placental region segmentation in US imaging (*143*).

Recent AI-based US research has actively developed models for predicting perinatal diseases. By combining multiple biomarkers, including UtA PI, with maternal clinical factors, machine learning algorithm with a fully connected neural network enhanced the performance of early PE screening (*144*). In addition, radiomics that extracts quantitative features from 2D/3D B-mode and Doppler US imaging, such as placental texture and blood flow, can support AI-based models for predicting perinatal disease risk. Radiomics-driven machine learning classifier using radial basis function support vector machine demonstrated strong early GDM prediction, with further improvement when combined with a Resnet-50 convolutional neural network and clinical variables (age and BMI) (*145*). An extreme randomized tree classifier using radiomics features extracted from placental B-mode US images achieved high accuracy in distinguishing normal from dysfunctional placentas. (*146*). Compared with other imaging modalities, US provides high repeatability, immediacy, and accessibility, facilitating data acquisition. This suitability for large-scale data collection offers distinct advantages in implementing advanced deep learning architectures, such as multimodal fusion networks, and in developing generalizable AI-based predictive models.

### Considerations toward clinical translation

**Fig. 9.**
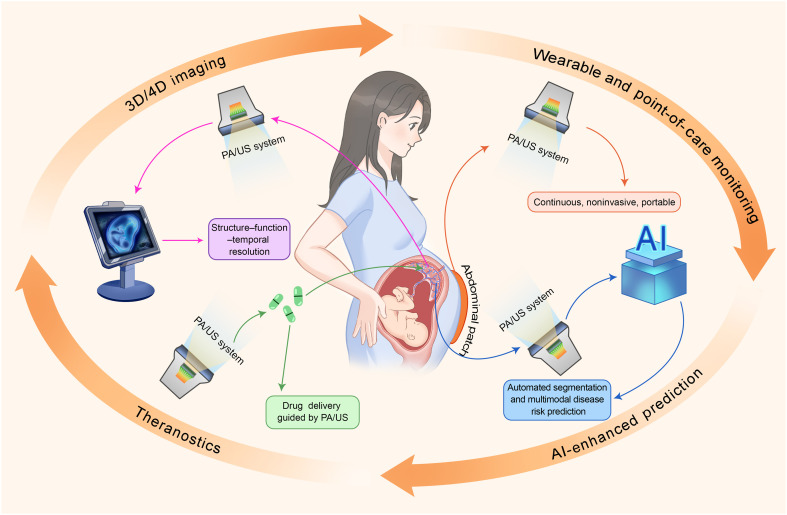
Future directions in placental imaging. 3D/4D imaging: Integrated visualization of placental structure and spatiotemporal function; Wearable and point-of-care devices: Continuous, portable, noninvasive monitoring of placental health; AI-enhanced predictive modeling: Automated analysis and multimodal risk prediction; Theranostics: PA/US-guided targeted therapy and real-time treatment assessment. This figure was created using Adobe Photoshop.

Overall, clinical translation of US/PA technologies should follow a systematic roadmap: benchtop validation, large animal safety testing, pilot human feasibility studies, and multicenter clinical trials. Establishing such stepwise protocols will ensure reproducibility, safety, and regulatory compliance, facilitating the eventual adoption of these technologies in clinical obstetrics (Fig. 9).

Ultrafast planewave US may require additional mechanical and thermal safety testing due to its kilohertz-range excitation of transducer elements and acquisition of hundreds of ensemble frames. Standardization of laser exposure limits would represent the greatest hurdle for PA placental imaging, as mentioned in the “Technological challenges and solutions toward clinical translation” section, requiring a strong driving force supported by clear evidence that PA imaging could address clinical unmet needs. The correlation between preclinical and clinical findings, upon the development of feasible animal models and reliable imaging systems, should be investigated to underscore the necessity of these imaging modalities and facilitate clinical translation.

## CONCLUSION

US and PA imaging offer previously unidentified opportunities for next-generation platforms that integrate structural and functional insights, opening horizons for early diagnosis, prognosis, and personalized monitoring of obstetric complications. In this review, we outlined the principles of US and PA imaging and their applications in both preclinical and clinical settings, highlighted key technical challenges and emerging solutions, and provided perspectives on future directions for advancing the field. With sustained efforts in large-scale clinical validation and standardization, these two modalities could ultimately redefine the paradigm of maternal and fetal care as indispensable tools for the future.
